# 2053: Evaluating impact of CTSA usage on research productivity outcomes

**DOI:** 10.1017/cts.2017.156

**Published:** 2018-05-10

**Authors:** Yue Zang, Tom Greene, Trent Matheson, Erin Rothwell

## Abstract

OBJECTIVES/SPECIFIC AIMS: In this study, we propose to investigate effectiveness of 2 core services provided by the Center for Clinical and Translational Science (CCTS), home for CTSA program in the School of Medicine at the University of Utah. METHODS/STUDY POPULATION: We will apply a longitudinal database of research and tenure track faculty (n>600) in the School of Medicine at the University of Utah from 2006 to 2016 to estimate the effect of initial usage of the biostatistics and clinical services cores of the University of Utah CCTS on the probability of (a) ≥1 peer reviewed publication, (b) external grant funding, and (c) academic promotion within 1, 2, and 3 years after the initial contact. We will apply a “new users” design (Hernan *et al*., *Epidemiology*, 2008; 19: 766–779) to compare the outcomes of faculty initiating use of the 2 CCTS cores Versus faculty without prior use of these cores in a series of cohorts defined by the calendar year of initial contract with the 2 cores, with covariate adjustment performed within each cohort to account for measured confounders. Separate outcome models will be specified for each cohort, but the statistical models will be fit to stacked augmented data sets which include the data from each cohort. Using the stacked data set, results will be pooled across each of the cohorts to increase statistical power. Robust sandwich estimates of standard errors will be used to account for the inclusion of multiple assessments for each faculty member. RESULTS/ANTICIPATED RESULTS: Estimates of the effect of initiation of new CTSA usage on academic productivity outcomes will be obtained, and provided in conjunction with sensitivity analyses to address the potential impact of uncontrolled confounding. DISCUSSION/SIGNIFICANCE OF IMPACT: The proposed evaluation strategy should overcome some of the biases inherent in typical metrics for effectiveness of CTSA programs, and will be applied to evaluate success of future initiatives.

